# Public-sector Maternal Health Programmes and Services for Rural Bangladesh

**DOI:** 10.3329/jhpn.v27i2.3326

**Published:** 2009-04

**Authors:** Malay Kanti Mridha, Iqbal Anwar, Marge Koblinsky

**Affiliations:** Public Health Sciences Division, ICDDR,B, GPO Box 128, Dhaka 1000, Bangladesh

**Keywords:** Maternal health, Maternal health services, Rural health services, Bangladesh

## Abstract

Achieving Millennium Development Goal 5 in Bangladesh calls for an appreciation of the evolution of maternal healthcare within the national health system to date plus a projection of future needs. This paper assesses the development of maternal health services and policies by reviewing policy and strategy documents since the independence in 1971, with primary focus on rural areas where three-fourths of the total population of Bangladesh reside. Projections of need for facilities and human resources are based on the recommended standards of the World Health Organization (WHO) in 1996 and 2005. Although maternal healthcare services are delivered from for-profit and not-for-profit (NGO) subsectors, this paper is focused on maternal healthcare delivery by public subsector. Maternal healthcare services in the public sector of Bangladesh have been guided by global policies (e.g. Health for All by the Year 2000), national policies (e.g. population and health policy), and plans (e.g. five- or three-yearly). The Ministry of Health and Family Welfare (MoHFW), through its two wings—Health Services and Family Planning—sets policies, develops implementation plans, and provides rural public-health services. Since 1971, the health infrastructure has developed though not in a uniform pattern and despite policy shifts over time. Under the Family Planning wing of the MoHFW, the number of Maternal and Child Welfare Centres has not increased but new services, such as caesarean-section surgery, have been integrated. The Health Services wing of the MoHFW has ensured that all district-level public-health facilities, e.g. district hospitals and medical colleges, can provide comprehensive essential obstetric care (EOC) and have targeted to upgrade 132 of 407 rural Upazila Health Complexes to also provide such services. In 2001, they initiated a programme to train the Government's community workers (Family Welfare Assistants and Female Health Assistants) to provide skilled birthing care in the home. However, these plans have been too meagre, and their implementation is too weak to fulfill expectations in terms of the MDG 5 indicator—increased use of skilled birth attendants, especially for poor rural women. The use of skilled birth attendants, institutional deliveries, and use of caesarean section remain low and are increasing only slowly. All these indicators are substantially lower for those in the lower three socioeconomic quintiles. A wide variation exists in the availability of comprehensive EOC facilities in the public sector among the six divisions of the country. Rajshahi division has more facilities than the WHO 1996 standard (1 comprehensive EOC for 500,000 people) whereas Chittagong and Sylhet divisions have only 64% of their need for comprehensive EOC facilities. The WHO 2005 recommendation (1 comprehensive EOC for 3,500 births) suggests that there is a need for nearly five times the existing national number of comprehensive EOC facilities. Based on the WHO standard 2005, it is estimated that 9% of existing doctors and 40% of nurses/midwives were needed just for maternal healthcare in both comprehensive EOC and basic EOC facilities in 2007. While the inability to train and retain skilled professionals in rural areas is the major problem in implementation, the bifurcation of the MoHFW (Health Services and Family Planning wings) has led to duplication in management and staff for service-delivery, inefficiencies as a result of these duplications, and difficulties of coordination at all levels. The Government of Bangladesh needs to functionally integrate the Health Services and Family Planning wings, move towards a facility-based approach to delivery, ensure access to key maternal health services for women in the lower socioeconomic quintiles, consider infrastructure development based on the estimation of facilities using the WHO 1996 recommendation, and undertake a human resource-development plan based on the WHO 2005 recommendation.

## INTRODUCTION

**Fig. 1.**
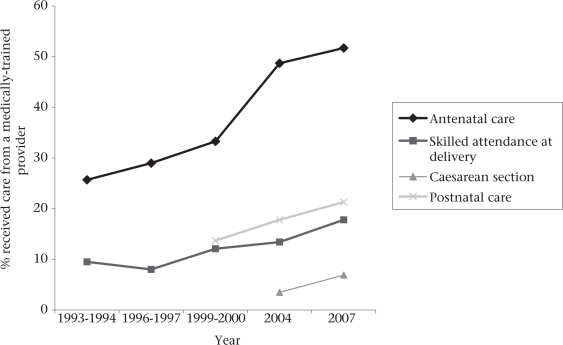
Antenatal and postnatal care, skilled attendance at birth, caesarean-section rate, Bangladesh, 1993-2007

**Fig. 2.**
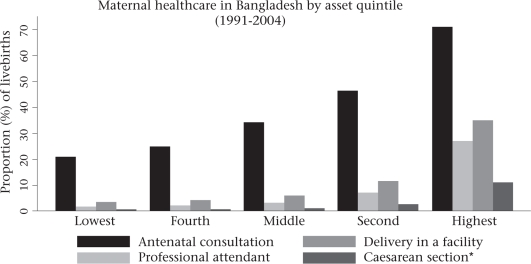
Access inequity in maternity-care services

Source: Demographic and Health Surveys; ∗From 1995 onwards

Bangladesh has made significant progress towards achieving the Millennium Development Goal (MDG) 5 target of 75% reduction in the maternal mortality ratio (MMR) between 1990 and 2015. Starting at 570/100,000 livebirths in 1990, there has been a 44% decline by 2001 to an MMR of 322 maternal deaths per 100,000 livebirths (1). Yet, this decline in the MMR does not correspond to improvements in the widely-used United Nations (UN) process indicators, e.g. skilled attendance at birth and population-based caesarean-section rate. According to the Bangladesh Demographic and Health Survey (BDHS) 2007, a medically-trained provider attended only 18% of births, 15% of deliveries took place in health facilities (2), and 6.9% had a caesarean section (Afsana K. Personal communication, 2008). Progress on the other process indicators, e.g. use of antenatal care and postnatal care, has been remarkable. The BDHS 2007 reported that 52% of mothers received at least one antenatal check-up, and 21% of mothers received at least one postnatal check-up from a trained care provider (Fig. 1). Despite this, inequities in the use of maternal health services are striking. The use of antenatal care, skilled birth attendants, institutional delivery, and caesarean section is substantially lower in the lower three socioeconomic quintiles (Fig. 2).

If the current trend in the MMR decline continues, the MMR in Bangladesh will reach 191/100,000 livebirths in 2015, although the target is 143/100,000 livebirths. To achieve this target, the rate of MMR decline per year must increase three-fold over the remaining seven years (3). To do so, ensuring adequacy and improving the use of maternal health services are key elements, along with other services that have already contributed to the reduced MMR, e.g. family planning and safe menstrual regulation. Improving the functionality of the health system is, thus, warranted, along with a projection of future needs.

In Bangladesh, both formal and informal sectors provide maternal health services. Non-public services are well-used and must be widely available, including private care providers (for-profit and not-for-profit) in the formal sector and in the non-formal sector, e.g. traditional birth attendants (TBAs) (52% of all institutional deliveries took place in non-governmental organization [NGO] and private health facilities in 2007 while 82% of all deliveries were with a TBA or other untrained care providers) (2). Despite their obvious importance, information on non-public-health facilities, manpower, and services is largely unavailable. Therefore, the focus of this paper is the evolution of the public-sector programme and its facilities, services, and manpower for improving maternal health. To judge the adequacy of the public-health system to provide maternal health services, we reviewed policy documents and available primary and secondary information. Facility and manpower needs to provide skilled care for all births are projected to 2015.

Bangladesh is a poor country in South Asia, with a projected population of 135.43 million in 2007, 74% of whom live in rural areas. The crude birth rate was 20.8 per 1,000 people, and the crude death rate was 5.8 per 1,000 people while the average literacy rate was only 46% (male 50%, female 42%). To serve this population, the country is divided into six divisions, such as Barishal, Khulna, Chittagong, Sylhet, Dhaka, and Rajshahi, and is further subdivided into 64 districts. At the district level, the population typically ranges from 1 to 2 million. Districts are divided into subdistricts (upazilas) in the rural areas or thanas in the metropolitan areas. The 508 upazilas/thanas are further divided into unions (4,484 in total). The population in the upazilas ranges from 0.2 to 0.45 million and in unions from 20,000 to 30,000.

This paper does not cover changes in the financing of the public-sector as it is extremely difficult to segregate resource allocations for maternal health from overall allocations.

## MATERIALS AND METHODS

To review the evolution of maternal health services with the national public-health system, we reviewed the existing government policy and strategic documents, such as five-yearly development plans (1973-2000); a three-yearly development plan (2003-2006); the Maternal Health Strategy of 2001; the Poverty Reduction Sector Paper of 2004; plan of the Health and Population Section Programme of (HPSP) 1998-2003; and the revised plan of the Health, Nutrition and Population Sector Programme (HNPSP) 2003-2010. Information available from the Management Information System (MIS) of the Ministry of Health and Family Welfare (MoHFW) was used for determining the availability of public-sector infrastructure, human resources, and services provided. Data generated from key-informant interviews with government and non-government officials were also helpful to understand the chronology of maternal health services in the country. Available secondary information, e.g. from BDHS reports, and the electronic data-bank developed by Partners in Health and Development (PHD), was also reviewed and interpreted.

Estimates of adequacy of infrastructure and human resources (HR) were based on standards proposed by the World Health Organization (WHO) modified slightly. In the 1996 recommendation for adequacy of health facilities for maternal care, the WHO proposed four basic EOC facilities and one comprehensive EOC facility for a population of 500,000 (4). In the World Health Report 2005, the recommendation was for one back-up facility (comprehensive EOC facility) for 3,600 births with three doctors, including one specialist in gynaecology and obstetrics; one birthing centre for every 1,750 births and one midwife or other professional per 175 births (5). In our calculations, we used one comprehensive EOC facility and four doctors, including one specialist in gynaecology and obstetrics, one anaesthetist, one paediatrician, and one general physician for every 3,500 births; we also used the 2005 WHO standard for the birthing centres and midwives/other professionals.

## RESULTS

### Policies and strategies  

In 1943, the importance of development of a primary healthcare system in Bangladesh (then part of India) was spelled out in the Bhore Committee report: Sir Joseph Bhore recommended an integrated form of healthcare services (preventive and curative) to be made available at the doorstep through health centres, ensuring equity and community participation. Based on the recommendation of the report, the Government of Pakistan designed a ‘rural health centre scheme' in 1961 to establish one rural health centre (RHC) in each thana (now upazila) to cover a population of 50,000 and one rural subcentre in each union to cover a population of 12,500. Establishment of RHCs and subcentres progressed slowly. Although the Government of Pakistan planned to establish 860 RHCs by 1970, only 230 RHCs were established by that time (6). The purpose of establishing the RHCs was to provide maternal, child and adult care; care for all illnesses, emergencies, and disabilities; epidemiological investigations and collection of vital statistics; control of communicable diseases; maintenance of eradication programmes and environmental sanitation; and implementation of family-planning programme, health education, and nutrition.

The health programme during which Bangladesh was part of Pakistan was the base on which Bangladesh built following the independence in 1971 but it was not until 2000 that the first Bangladesh National Policy on Health was formulated. Before the endorsement of the policy, the health programmes of Bangladesh were guided by five-yearly plans described in Table 1.

**Table 1.** Major plans of the Bangladesh Government and relevance to maternal health (7)

Plan	Highlights relevant to maternal health
First 5-year plan (1973-1978)	• Establish 31-bed hospitals in ‘Thana Health Complexes' in rural thana (now upazila)
	• Develop and expand training facilities for doctors, nurses, and paramedical staff
	• Increase the production of essential drugs
	• Develop a national population policy
	• Set up a separate family-planning wing of the Ministry of Health and Family Welfare
	• Set up family welfare visitor (FWV) training institutes and organize training of FWVs
Second 5-year plan (1980-1985)	• Accept primary healthcare for all as the strategy to reach the goal ‘Health for All by 2000’
	• Set the target for completion of setting up Thana Health Complexes in each rural thana (now upazila) and establish one Union Health and Family Welfare Centre' in each union by 1985
	• Start menstrual regulation as a method for family planning
	• Train traditional birth attendants
Third 5-year plan (1985-1990)	• Continue with the strategy ‘primary healthcare' for all
	• Integrate maternal and child healthcare and family-planning care
Fourth 5-year plan (1990-1995)	• Continue with the strategy for primary healthcare for all
	• Intersectoral collaboration
	• Integrate maternal and child healthcare, family-planning care, nutrition care, and health education
Fifth 5-year plan (1997-2002)	• Health and Population Sector Programme
	Implement essential service package (reproductive healthcare, child healthcare, communicable disease control, limited curative care; and behaviour change communication) in all facilities
	• Set up 13,000 community clinics—1 per 6,000 people to provide one-stop services
	• Decentralize the process for programme planning, strategy formulation, and resource mobilization
	• Ensure equity of access to services
	• Unify health and family-planning wings of the Ministry of Health and Family Welfare to improve programme management and service-delivery
	• Develop a national health policy
	• Develop a national strategy for maternal health
	• Commence a community-based skilled birth-attendant programme
First 3-year plan (2003-2006)	• Health, Population and Nutrition Sector Programme
	• Provide essential service-delivery (reproductive health, child health, limited curative care, urban health services, healthcare, waste management, support services, and coordination)
	• Upgrade facilities, train manpower, ensure equipment and supplies, and further develop referral linkages
	• Implement Women-Friendly Hospital Initiative in tertiary and secondary-level healthcare facilities
	• Implement Demand-Side Financing Pilot: Maternal Health Voucher Scheme in 21 upazilas
	• Separate health and family-planning wings of the Ministry of Health and Family Welfare

### Development of infrastructure and service provisions

At independence in 1971, Bangladesh inherited one postgraduate medical institute, eight medical college hospitals, along with a small number of clinics, health centres, and dispensaries. Since then, setting up of health centres has been emphasized in the health-sector plans based on the five-yearly national plans. By 2006, under the authority of both Health Services and Family Planning wings of the bifurcated MoHFW, Bangladesh had grown substantially as shown in Table 2 (8,9). The number of beds in these facilities increased in 2006 to 0.29 per 1,000 people, including 2,000 in postgraduate medical institutes, 5,771 in medical college hospitals, 4,200 in district hospitals, 748 in Maternal and Child Welfare Centres (MCWCs), 12,672 in Upazila Health Complexes (UHCs), and 178 in RHCs.

**Table 2.** Growth of public-health infrastructure providing maternal health services since 1971 (7-9)

Health facility	No. of health facilities						
		Base year (1971)	End of 1^st^ FYP (1978)	End of 2^nd^ FYP (1985)	End of 3^rd^ FYP (1990)	End of 4^th^ FYP (1995)	End of 5^th^ FYP (2002)	End of 1^st^ TYP (2006)
Postgraduate medical institute	1	3	3	5	5	5	7
Medical college hospitals	8	8	10	10	13	13	14
District hospitals (Health Services wing)	-	37	59	59	59	59	59
Maternal and Child Welfare Centres (Family Planning wing)	91	93	96	96	96	96	97
Upazila Health Complexes (Health Services wing)	151 (RHC)	253 (UHC+RHC)	346	352	365	395	407
Union subcentre/RDs (Health Services wing)	1,450	1,752	1,275 (USC and RD merged)	1,310	1,362	1,362	1,362
Union Health and Family Welfare Centre (Family Planning wing)	-	1,275	2,329	Data not available	2,706	3,275	3,478

FYP=Five-year plan; RD=Rural dispensary; RHC=Rural health centre; TYP=Three-year plan; UHC= Upazila Health Complex; USC=Union subcentre

Key family-planning and maternal health services available in Bangladesh include antenatal care (ANC), menstrual regulation (MR), family-planning services, postabortion care (PAC), basic EOC, comprehensive EOC, postnatal care (PNC), etc. However, not all types of maternal and family-planning services are available from all the health facilities. For example, comprehensive EOC services are available in a few UHCs, most MCWCs, most district hospitals, all medical college hospitals, and one postgraduate institute. A brief description of maternal and family-planning service provision in the various public-sector facilities is given in Table 3.

**Table 3.** Provision of services and human resources by type of health facility, Bangladesh, 2007

Type of health facility and approximate population served	Location	Maternal health service providers	Provision of maternal health services, 2007
Health Services wing of MoHFW			
Medical college (10-15 million)	3 in capital city, 5 in 5 other divisions, 6 at district level	Doctors (professor, associate professor, assistant professor, consultants, registrar, assistant registrar, indoor medical officer, trainees, interns, anaesthesiologists), nurses (senior staff nurse, staff nurse, student nurse), medical technologists, pharmacists, ward boy, *Aya*	ANC, CEOC, BEOC, PNC, MR, AC, FPS
District hospital (1-2 million)	Districts	Doctors (senior and junior consultants, resident physician, resident medical officer, medical officer, anaesthesiologists), nurses (senior staff nurse, staff nurse, student nurse), medical technologists, pharmacists, ward boy, *Aya*	ANC, CEOC, BEOC, PNC, MR, AC, FPS
Upazila Health Complexes (Health Services wing) (0.2-0.45 million)	Upazila	Specialist doctors, medical officer, nutritionist, medical assistant, pharmacists, medical technologists, nurses, assistant nurses, health assistants, ward boy, *Aya*	ANC, CEOC in 77 of 132 (targeted), BEOC in most, PNC
Union subcentre/rural dispensaries (20-30 thousand)	Union	Medical officer, medical assistant, pharmacist	ANC, PNC
Family Planning wing of MoHFW			
Maternal and Child Welfare Centre (1-2 million in districts, 0.2-0.45 million in upazilas, 20-30 thousand in unions)	62 in districts, 12 in upazila, 23 in unions	Medical officer, family welfare visitor, pharmacist, family welfare assistants, nursing attendants, ward boy, *Aya*	ANC, CEOC (in 62 district-level MCWCs), BEOC, PNC, MR, AC, FPS
Upazila Health Complexes (Family Planning wing) (0.2-0.45 million)	Upazila	Medical officer, family welfare visitor, medical assistant, pharmacist, family welfare assistants, nursing attendants	MR, FPS
Union Health and Family Welfare Centre (20-30 thousand)	Union	Medical officer, medical assistant, family welfare visitor, pharmacist, *Aya*	BEOC available in a few of 1,495 (targeted), ANC, PNC, FPS

AC=Abortion care; ANC=Antenatal care; BEOC=Basic essential obstetrics care; CEOC=Comprehensive essential obstetrics care; FPS=Family-planning services; MCWCs=Maternal and Child Welfare Clinics; MR=Menstrual regulation; PNC=Postnatal care

### Health workforce for maternal healthcare

In the public sector, 63% of the total workforce is involved in providing health services (7). Human resources for maternal healthcare include specialist doctors, general physicians, nurses, medical assistants, pharmacists, medical technologists, family welfare visitors, community-based skilled birth attendants, family welfare assistants, and health assistants. Apart from health assistants and family welfare assistants who provide care at the doorstep of homes, others are based at facilities (Table 3). There is no estimate of the proportion of general physicians providing maternal healthcare. Table 4 provides numbers, education, and training of different types of health providers for maternal healthcare. 

**Table 4.** Training and education of various types of maternal healthcare providers, Bangladesh, 2006

Type of healthcare provider	Annual intake	Total no. (2006)	Requirement for entry	Education and training	Key maternal health services provided
Specialist in gynaecology and obstetrics	143	1,070	MBBS	Minimum 4 years of training and education for MS and FCPS degrees	BEOC, CEOC, ANC
				2 years of training and education for diploma	
Anaesthesiologist	152	860	MBBS	Minimum 4 years of training and education for MD and FCPS	Anaesthesia
				2 years of training and education for diploma	
General physicians	3,200 (1,475 in government)	44,632	12 years of schooling	5 years of training on medicine, surgery, gynaecology and obstetrics	ANC, BEOC, PNC, MR, AC, FPS
				1-year internship in medicine, surgery, gynaecology and obstetrics wards	
Nurses/midwives	1500 (1,020 in government)	40,040	10 years of schooling	Training on nursing for 3 years	ANC, BEOC, PNC, MR, AC, FPS
				Midwifery training for 1 year
Medical assistants	240	4,348	10 years of schooling	3 years of training on treatment of common disorders	ANC, BEOC, PNC, FP
Family welfare visitor	None since 1995	4,286	10 years of schooling	18 months of training on MCH, family planning, and contraception	ANC, BEOC, PNC, MR, AC, FPS
Family welfare assistant	None since 1995	23,500	10 years of schooling	30 days of training on family planning	ANC, PNC FPS
Community-based	1,000	1,500	FWA or Female Health Assistant	6 months of training on BEOC and ENC	ANC, BEOC, PNC, FPS
Health assistant	None since 2004	21,000	10 years of schooling	3 months of training on limited preventive and curative care, immunization	Tetanus toxoid

AC=Abortion care; ANC=Antenatal care; BEOC=Basic essential obstetric care; CEOC=Comprehensive essential obstet rics care; ENC=E ssential newborn care; FCPS=Fellow of College of Physicians and Surgeons; FPS=Family-planning services; MCH= Maternal and child health; MD=Doctor of Medicine; MR=Menstrual regulation; MS=Masters of Surgery; PNC=Postnatal care

### Organization of maternal health services

Maternal health services in Bangladesh are delivered through both community-based and facility-based approaches. The emphasis on community-based approaches was initiated in 1978 to reach the goal—‘Health for All by the Year 2000'. The facility-based approach gained momentum in 1987 only after the commencement of the safe motherhood initiative.

### Community-based approach

Extension of the health services infrastructure to the community level became part of the population-control and primary healthcare policies and strategies of the 1970s and 1980s. A 1975 national policy for population control supported the employment of thousands of full-time female field workers (FWAs) in the Family Planning wing of the MoHFW. The number of FWAs increased to 23,500 in 1989 to allow household-visits of once every two months in a catchment area of 4,000 people. The FWAs implemented the community-based distribution of family-planning methods, such as oral pills and condoms, along with referral for other methods.

Integration of maternal and child healthcare with family planning was specified in the five-year plans since 1975. The FWAs were instructed to provide ANC and refer high-risk pregnancies, alongside their family-planning tasks. This integration was aimed at establishing a maternal health and family-planning service-delivery system from household to the subdistrict level, although the two wings of the Ministry—Health Services and Family Planning—remained separate. However, targets for the performance of FWAs focused on family planning, and hence, the FWAs worked primarily as family-planning agents. 

Interventions for maternal health during 1980-1990 (second and third 5-year plan periods) again tried to piggyback on the existing personnel and systems. Tetanus toxoid (TT) vaccination was provided under the Expanded Programme on Immunization (EPI) of the Health Services wing and provided jointly by the FWAs (Family Planning wing staff) and HAs (Health Services wing staff). During the 1980s, the numbers of HAs increased to 21,000 to ensure one HA per 4,000-5,000 people. The HAs were also responsible for the delivery of primary healthcare, although their role primarily focused on immunization of mothers and children. Other maternal interventions were strongly guided by the WHO focus at that time: Screening of high-risk pregnancies was done through antenatal check-ups by the FWVs (Family Planning wing), and TBAs were trained in all districts. By 1994, there were 52,075 trained TBAs. The FWVs were providing facility-based ANC, PNC, and basic EOC from the union-level HFWCs. Although their training included development of midwifery skills, the focus of services by the FWVs gradually shifted to family planning as they were recruited by the Family Planning wing of the MoHFW and monitored against their performance in terms of family-planning services.

In 2001, the Bangladesh Maternal Health Strategy recommended a community-based skilled birth attendant (CSBA) strategy to complement the facility-based strategy described below. The document set a target of one six-month-trained CSBA for 6,000-8,000 people. The Obstetrical and Gynaecological Society of Bangladesh and the MoHFW, in collaboration with WHO and the United Nations Population Fund, started implementing a pilot project in 2001, which was aimed at educating and training the existing FWAs and female HAs with skills needed to deliver ANC, basic EOC, and PNC services along with essential newborn care (ENC) at the household level. The CSBA training programme was gradually expanded to 28 of the 64 districts of the country. Up to June 2007, about 2,500 FWAs and female HAs had completed their training and were deployed to provide home-based skilled delivery care, in addition to their previous family-planning responsibilities. Other than the CSBA programme and the one-year midwifery training of nurses, Bangladesh has no certified midwifery cadre.

### Facility-based approach

Since the beginning of the Safe Motherhood Initiative in 1987, reduction of maternal mortality has played a central role in health policies and operational plans as reflected in the fourth five-year development plan (1990-1995). Beginning in 1994, the MoHFW followed the emergency obstetrics care (EmOC) approach and focused on renovating and upgrading the existing facilities to provide EmOC services. In 2003, the target set by the HNPSP was to provide comprehensive EOC services from all 14 medical college hospitals, 59 district hospitals, 64 of the 96 MCWCs, and 132 of the 407 UHCs. Its main intervention elements were construction and renovation, logistics and equipment, and human-resource development (HRD). In 2006, the Government also decided to upgrade 1,495 of the 3,478 union-level HFWCs to provide basic EOC.

### Governance of maternal health services

As mentioned earlier, the MoHFW has a bifurcated structure from the top down to the grassroots level, with one cadre of workers engaged in family-planning activities and the other in health activities. This bifurcated structure was created in 1974 when the Population Control and Family Planning Division was established within the then Ministry of Health and Population Control (10). The health-sector reform initiated in 1996 attempted to unite these two wings of the MoHFW but failed to sustain the unification after 2001.

The MoHFW is responsible for the overall coordination of the implementation of the current programme—HNPSP—with contributions of Development Partners to the Programme. The MoHFW is accountable for making programme implementation adhere to the procedures and guidelines of the Government of Bangladesh (GoB) and Development Partners, and for overall transparency and accountability in using HNPSP funds (11). 

**Fig. 3.**
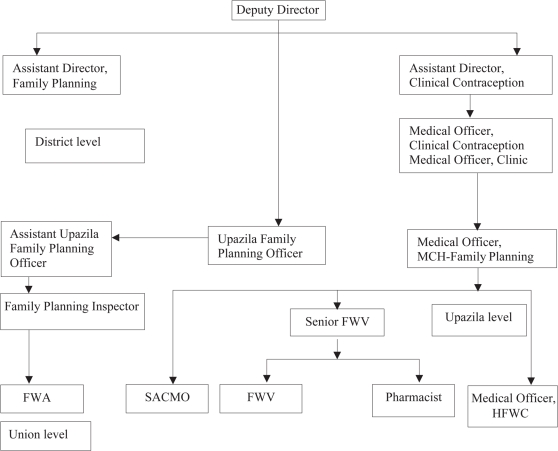
Organogram of Directorate of Family Planning: district level and below (2005)

Source: Perry HB. 2005 (10) FWA=Family Welfare Assistant; FWV=Family Welfare Visitor; HFWC=Health and Family Welfare Center; MCH=Mother and child health; SACMO=Sub-Assistant Community Medical Officer

With policy and administrative guidance from the MoHFW, the Line Directors in the Director Generals' offices implement the 38 programmes of the HNPSP through a centralized approach. Director-Primary Health Care and Director-Hospital and Clinics from the Health Services wing (Directorate General of Health Services) and Director-MCH Services and Director-Clinical Contraceptive Service Delivery from the Family Planning wing, are the key persons responsible for planning and implementation of the maternal health programme in Bangladesh. Through the divisional offices of the Director-Health and Director-Family Planning in the six divisions of Bangladesh, the respective directors provide administrative guidance to the district-level health and family-planning authorities. Figure 2 and 3 depict the organizational structures of health and family-planning offices at the district level and below.

**Fig. 4.**
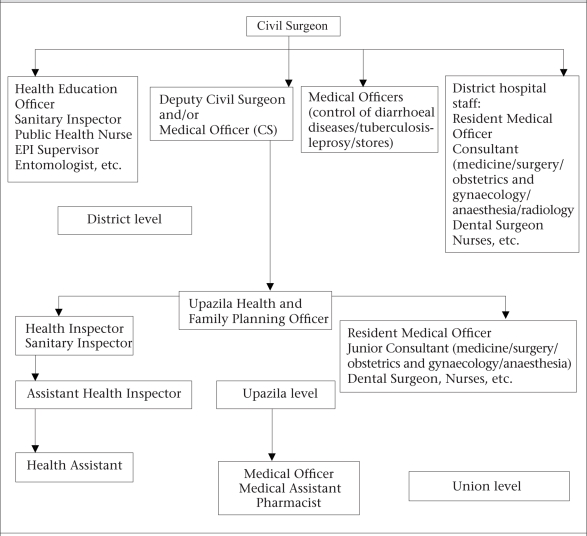
Organogram of Directorate of Health Services: district level and below (2005)

Source: Perry HB. 2005 (10) CS=Caesarian section; EPI=Expanded Programme on Immunization; TB=Tuberculosis

The organogram of the Family Planning wing at district and below clearly depicts a dual chain of authority through the Upazila Family Planning Officer (UFPO) and Medical Officer-MCH. The Medical Officer-MCH has no supervisory responsibility for the community-level FWAs, and the UFPO has no supervisory authority over the activities of the union-level FWVs. As a result, coordination of activities between FWVs and FWAs in a given union becomes difficult. Moreover, all the FWAs are female and all family-planning inspectors are male, who never worked as FWAs (10). As a consequence, they are not able to supervise the FWAs in their day-to-day home-visit activities.

On the other hand, the Health Services wing is free from the dual chain of supervision at the district level and below. The assistant health inspectors (AHIs) and the HAs supervise and, in contrast to the family-planning inspectors, the AHIs have worked in their positions for a number of years.

As mentioned earlier, staff of the Directorate General of Health Services (DGHS) and Directorate General of Family Planning (DGFP) work independently even when working in the same geographical area. The HAs have a little contact with the FWAs, and the AHIs have a little or no contact with the FPIs. The same is true for staff of both the wings at the upazila, district, division and national levels. There is no sharing of performance and management information at any level, except at the national level—MoHFW. Although this bifurcation was necessary during the 1980s and 1990s for rapid progress of the family-planning programme, it has also led to duplication in management, service-delivery of staff, inefficiencies as a result of these duplications, and difficulties of coordination at all levels. 

### Adequacy of basic EOC and comprehensive EOC facilities and human resources

Based on the WHO 1996 recommendation of 1 comprehensive EOC facility for 500,000 people and 1 basic EOC facilities for 125,000 people, Table 5 shows that 82% of needed comprehensive EOC facilities existed as of 2007 (1 postgraduate medical institute, 14 medical college hospitals, 62 MCWCs, 59 district hospitals, 85 of 132 upgraded UHCs) in the public sector of Bangladesh. However, there is a wide variation among the six divisions: Rajshahi division has more facilities than set by the WHO standard whereas Chittagong and Sylhet divisions have only 64% of their need for comprehensive EOC facilities. The numbers of basic EOC facilities are not sufficient in any division. If we estimate that all comprehensive EOC facilities and all UHCs can provide basic EOC, only 50% of the need for basic EOC facilities is fulfilled. However, if all the 1,495 upgraded HFWCs start providing basic EOC services as planned in the HPSP 2003-2006, the level of basic EOC facilities per the WHO recommendation will be reached.

**Table 5.** Adequacy of BEOC and CEOC facilities (2007) based on WHO recommendations, 1996 and 2005

Division	WHO 1996 recommendation				WHO 2005 recommendation			
	No. of CEOC facilities needed	No. (%) of CEOC facilities existing	No. of BEOC facilities needed	No. (%) of BEOC facilities existing	No. of CEOC facilities needed	No. (%) of CEOC facilities existing	No. of BEOC facilities needed	No. (%) of BEOC facilities existing
Barisal	18	18 (100)	75	49 (65)	69	18 (26)	138	49 (36)
Chittagong	53	34 (64)	221	108 (49)	204	34 (17)	408	108 (26)
Dhaka	85	59 (69)	357	139 (39)	330	59 (18)	660	139 (21)
Khulna	46	42 (91)	193	63 (33)	179	42 (23)	358	63 (18)
Rajshahi	52	57 (110)	216	147 (68)	199	57 (29)	398	147 (37)
Sylhet	17	11 (64)	72	37 (51)	67	11 (16)	134	37 (28)
Total	271	221 (82)	1,083	543 (50)	1,048	221 (21)	2,096	543 (26)

BEOC=Basic essential obstetric care; CEOC=Comprehensive essential obstetric care; WHO=World Health Organization

**Table 6.** Adequacy of maternal healthcare providers, Bangladesh, 2007

Division	Total population in 2007	Total births in 2007	No. of doctors needed for maternal healthcare	% of existing doctors needed for maternal healthcare	No. of nurses/midwives needed for maternal healthcare	% of existing nurses needed for maternal healthcare
Barishal	8,915,893	24,1315	276		1,379	
Chittagong	26,373,436	71,3816	816		4,079	
Dhaka	42,638,973	1,154,054	1319		6,595	
Khulna	23,091,116	624,977	714		3,571	
Rajshahi	25,778,843	697,722	797		3,987	
Sylhet	8,634,615	233,702	267		1,335	
Total	135,432,877	3,665,586	4189	9	20,946	40

Shaded columns indicate that division-level estimates of existing doctors/nurses are not available

The estimate based on the 2005 WHO recommendation, i.e. 1 comprehensive EOC facility and 2 basic EOC facilities for 3,500 births, gives a different picture. As per this recommendation, there is a need for 1,048 comprehensive EOC facilities (nearly five times the existing total) and 2,096 basic EOC facilities (nearly four times the existing total). The need for basic EOC facilities will be almost met if all the upgraded HFWCs provide basic EOC services. Estimates based on both the recommendations show that the met need of basic EOC and comprehensive EOC facilities is the highest in Rajshahi division and the met need of comprehensive EOC facilities is the lowest in Sylhet division.

Using the 2005 WHO recommendation, the estimation of required human resources (doctors and nurses/midwives) has also been reached. Bangladesh had 44,632 registered doctors in 2006; only 9% of them are needed to provide back-up support in comprehensive EOC facilities. Bangladesh is among the few countries in the world where there are nearly as many doctors as nurses and midwives. Based on the WHO guideline, it is estimated that 40% of the existing 40,040 nurses in 2006 are needed for maternal healthcare in Bangladesh in both basic EOC and comprehensive EOC facilities. Due to the unavailability of data for human resources, it was not possible to generate division-level estimates for the proportion of the existing doctors and nurses needed for maternal healthcare.

Using the WHO 1996 and 2005 recommendations, the projected need for basic EOC and comprehensive EOC facilities and human resources for maternal healthcare in 2015 was estimated. Based on the projected population and livebirths in 2015 of 152 million and 4.1 million respectively, 304 comprehensive EOC and 1,220 basic EOC facilities will be needed at that time to cover births and provide back-up support for mothers (WHO 1996 recommendation). On the other hand, the WHO 2005 recommendation projects the need for 1,180 comprehensive EOC and 2,358 basic EOC facilities in 2015. Comparing the information presented in Table 5 and 7, it can be deduced that there is a need to increase the number of basic EOC and comprehensive EOC facilities by 38% and 125% respectively as per the 1996 WHO recommendation over the next seven years. The WHO 2005 recommendation projects the need for a 334% and 434% increase in the number of basic EOC and comprehensive EOC facilities respectively. In 2015, 4,720 (531 more than the need in 2007) doctors and 23,595 nurses/midwives (2,649 more than the need in 2007) will be needed in those facilities to cover births according to the WHO 2005 recommendation.

**Table 7.** Need for BEOC and CEOC facilities in 2015, Bangladesh

Division	Total population in 2015	Total births in 2015	WHO 1996 recommendation		WHO 2005 recommendation			
			No. of CEOC facilities needed	No. of BEOC facilities needed	No. of CEOC facilities needed	No. of EOC facilities needed	No. of doctors needed for maternal healthcare	No. of nurses/midwives needed for maternal healthcare
Barishal	10,043,687	271,839	20	80	78	155	311	1,553
Chittagong	29,709,480	804,108	59	238	230	459	919	4,595
Dhaka	48,032,487	1,300,033	96	384	371	743	1,486	7,429
Khulna	26,011,971	704,032	52	208	201	402	805	4,023
Rajshahi	29,039,676	785,979	58	232	225	449	898	4,491
Sylhet	9,726,830	263,263	19	78	75	150	301	1,504
Total	152,564,132	4,129,255	304	1,220	1,180	2,358	4,720	23,595

BEOC=Basic essential obstetric care; CEOC=Comprehensive essential obstetric care; WHO=World Health Organization

## DISCUSSION

Maternal health services in Bangladesh evolved over time, guided by global and national policies and plans and internal politics. During 1973-1978 and onwards, the national population policy guided the direction and changes in family-planning services. The goal ‘Health for All by the Year 2000' later was a major factor for the development of healthcare plans but maternal health received limited priority. During the early 1980s, the Government followed the WHO strategies at the time, primarily focused on training of TBAs to ensure clean delivery at home. The Government also started the MR programme during this time in response to the wartime atrocities of 1971.  It was only with the Safe Motherhood Initiative starting in 1987 that maternal health became a major focus—with the emergency obstetric approach the key to both policy and programme efforts. 

Integration of maternal health with family-planning care started receiving attention with the advent of the 1987 safe motherhood movement. The most obvious example of this was delivery of services through an integrated approach for maternal health and family planning at the union level and below during 1997-2002, when the Government unified the Health Services and Family Planning wings, but unification was short-lived. In 2003, with the start of the first phase of the HNPSP (2003-2006), the Family Planning and Health Services wings were separated again, and the community clinics were closed. Their separation deterred further integration and left grassroots-level workers of both the wings in a state of confusion about their roles and responsibilities.

Despite these policy shifts, development of the infrastructure continued though not in any uniform pattern. Under the Family Planning wing, although the number of MCWCs remained static, new services were integrated, including caesarean-section surgery. It should be noted that most MCWCs offering comprehensive EOC are situated at the district level where district hospitals under the Health Services wing also provide comprehensive EOC, making comprehensive EOC very accessible in district towns. This is true even in places with fewer comprehensive EOCs than the WHO standard (e.g. Sylhet and Chittagong). The rural areas, however, remain without adequate coverage of comprehensive EOC. To rectify this and reach rural populations, the Health Services wing has been upgrading rural UHCs to provide comprehensive EOC since 1987. Although the plan was to upgrade 132 UHCs (26% of upazilas/thanas), the initiative could not be implemented per plan primarily due to the unavailability of skilled manpower and an inefficient retention strategy. As a result, the number of functional UHCs providing comprehensive EOC in rural areas hovered around 70-80 in 2007 (17-20% of all UHCs).

The problem of availability of skilled manpower, especially obstetricians and anaesthetists, has prevailed since 1971. In 2006, there were 873 specialists in gynaecology and obstetrics in the country. If 15% of women need treatment for complications during childbirth, one specialist in gynaecology and obstetrics would need to treat about 514 cases of childbirth-related complications per year. Considering the multiple involvements of these specialists in other work, their numbers are obviously very low. Moreover, there are no data on how many of them are active in providing services. To address this issue, the Government initiated a one-year training for medical graduates (MBBS) in each of these specialties. By 2007, 156 medical graduates received training on obstetrics. At the same time, 509 nurses also received training on EOC (Islam N. Personal communication, 2008). Despite these initiatives, the Government failed to develop enough skilled manpower, deployed, and retained comprehensive EOC at the upazila level. Poor selection of trainees, non-recognition by the professional associations of the one-year EOC and anaesthesia training as postgraduate training, inefficient deployment, and lack of incentives for working in rural areas, are a few factors limiting success of the comprehensive EOC programme of the Health Wing (Islam KS. Personal communication, 2008).

The roles of medical assistants and FWVs at the union-level clinics, and FWAs at the community level, are also vital in providing maternal healthcare. However, most medical assistants being male, they seldom receive further training on maternal health services. Recruitment and training of FWVs and FWAs were stopped in 1994. Due to attrition of FWVs and FWAs, the existing workforce is failing to reach all those who need their services. The recent reduction in the contraceptive prevalence rate for modern methods and household-visits by FWVs or FWAs as reported by the BDHS 2007 substantiates this statement. The community skilled birth attendant programme, initiated in 2001, whereby the FWAs receive training to become CSBAs, has not been able to fill the gap nor will it be able to by 2015 given the slow production of CSBAs and relatively-low usage by women for delivery. In 2006, it was calculated that, if Bangladesh continues to develop CSBAs at the current rate and deploy them in the community, the CSBAs will be able to cover only 5% of all births in 2015 (12).

Although Bangladesh has undertaken a two-pronged approach (community- and facility-based) to ensure maternal health services and has an extensive rural healthcare and family-planning service-delivery infrastructure, services have not reached most people who need them. Only 18% of women accessed skilled care at delivery in 2007 (2). Five years after the initiation of the CSBA programme, the CSBAs conducted only 0.1% of deliveries (2). The use of ANC, SBAs, institutional delivery, and caesarean section is substantially lower in the three lower socioeconomic quintiles (13). To address the equity issue, the Government piloted a demand-side financing scheme (popularly known as the maternal health voucher programme) in two upazilas in 2007 and has scaled up to 33 upazilas (6% of upazilas/thanas) in 2008. The scheme provides financial incentives to health service providers and mothers for providing and using selected maternal health services.

The paper has obvious limitations as it is based on information from the public sector only. A comprehensive programme to improve the maternal health status in Bangladesh cannot ignore the contributions of NGOs, for-profit-private sector, and informal service providers, yet the lack of available data on facilities and manpower in these sectors precludes their present inclusion.

### Conclusion

Due to the poor coverage of skilled care at delivery and inequity in the use of maternal health services, Bangladesh needs to revisit her maternal health strategy, especially for rural areas. One option is to recruit more FWAs, train, and deploy them as CSBAs along with FWVs in the upgraded union-level HFWCs to provide facility-based delivery care close to women. Facility-based delivery care is more efficient than home-care and can substantially increase the coverage of skilled care at delivery. Referral mechanisms, now non-existent, need to be put in place. The programme to train MBBS doctors to perform caesarean sections and provide anaesthesia needs to be strengthened to increase the numbers of functional upazila-level comprehensive EOC facilities. As retention of these skilled providers in rural areas is a major issue, strategies to address the retention problem need to be formulated, perhaps adapted from those used in Gujarat or Tamil Nadu (14-17).

At the same time, strategies to ensure access of the lower socioeconomic quintiles to key maternal health services, including caesarean delivery, need to be in place throughout the country. The maternal voucher scheme recently initiated should be followed closely. At antenatal care visits, increased demand for services, especially for those with complicated deliveries, needs to be stimulated through communications aimed at recognition of danger signs and where to go for appropriate care.

Further improvement of the governance of Health Services and Family Planning wings is necessary to make progress towards achieving the targets of health-related MDGs. The Health Services and Family Planning wings have similarities in maternal health-service provisions. Functional coordination between these wings to organize the delivery of services in the same geographic locations and to share information on the key maternal health indicators to review progress is required to improve efficiency of both the wings. At the same time, the NGOs and for-profit-private clinics delivering maternal health services should be involved in the overall planning and implementation of the maternal health programmes. It is expected that coordination will also help effectively allocate and use resources, and identify and reach underserved areas.

The varying recommendations of WHO have raised further difficulties in planning for the development of human resources and infrastructure. Given that Bangladesh is very densely populated and distances are not far from communities to upazila centres, the Government may consider the development of the infrastructure based on the estimation of facilities using the WHO 1996 recommendation. The human resource-development plan should, however, be based on the WHO 2005 recommendation so that there are adequate numbers of healthcare providers for maternal health.

## ACKNOWLEDGEMENTS

The Department for International Development (DFID) funded this work through a multicountry project titled “Case studies for safe motherhood: Learning from South Asian Programmes”.

The authors extend their thanks to the staff members of the Reproductive Health Unit of the Public Health Sciences Division of ICDDR,B for support to the project. The authors express their gratitude to Dr. Nazrul Islam, Deputy Programme Manager of Reproductive Health Programme of the Directorate General of Health Services and Dr. Khaled Shamsul Islam, Senior Assistant Chief of the Ministry of Health and Family Welfare, Government of Bangladesh, for the relevant information. The authors also express their gratefulness to the study participants for their active participation throughout the project.
